# Effects of local network topology on the functional reconstruction of spiking neural network models

**DOI:** 10.1007/s41109-017-0044-1

**Published:** 2017-07-18

**Authors:** Myles Akin, Alexander Onderdonk, Yixin Guo

**Affiliations:** Department of Mathematics, Drexel University, Chestnut Street, Philadelphia, USA

**Keywords:** Multiplex, Dyiadic subgraph, Triadic subgraph, Functional neural network, Structural neural network, Network information flow

## Abstract

The representation of information flow through structural networks, as depicted by functional networks, does not coincide exactly with the anatomical configuration of the networks. Model free correlation methods including transfer entropy (TE) and a Gaussian convolution-based correlation method (CC) detect functional networks, i.e. temporal correlations in spiking activity among neurons, and depict information flow as a graph. The influence of synaptic topology on these functional correlations is not well-understood, though nonrandom features of the resulting functional structure (e.g. small-worldedness, motifs) are believed to play a crucial role in information-processing. We apply TE and CC to simulated networks with prescribed small-world and recurrence properties to obtain functional reconstructions which we compare with the underlying synaptic structure using multiplex networks. In particular, we examine the effects of the surrounding local synaptic circuitry on functional correlations by comparing dyadic and triadic subgraphs within the structural and functional graphs in order to explain recurring patterns of information flow on the level of individual neurons. Statistical significance is demonstrated by employing randomized null models and *Z*-scores, and results are obtained for functional networks reconstructed across a range of correlation-threshold values. From these results, we observe that certain triadic structural subgraphs have strong influence over functional topology.

## Introduction

Abstract networks have been used to study complex interactions between many different types of actors (Boccaletti et al. 2006). These actors and interactions represent a range of applications including social connections among people (Holland and Leinhardt 1970; Haynie 2002; Gleiser and Danon 2003), games and economics (Kim et al. 2002; Ebel and Bornholdt 2002; Holme et al. 2003), protein-protein interactions (Barabasi and Oltvai 2004; Maslov and Sneppen 2002; Jeong et al. 2001) and cellular signaling (Weng et al. 1999; Bhalla and Iyengar 1999; StoŻer et al. 2013). In neuroscience, abstract complex networks have been implemented to model (a) structural and (b) functional neural networks. The former model the structure of neural tissue by representing either brain regions at the macroscopic level or individual neurons and synapses at the microscopic scale (Downes et al. 2012; Shimono and Beggs 2015; Sporns 2010). Structural networks in this paper represent the microscopic level as much of the brain’s information-processing and storage capacity is thought to arise from its synaptic connections and the structure they determine (Sporns 2010). It is therefore necessary to identify important features of this structure and their roles in information-processing and storage.

Functional networks, by contrast, are constructed from correlations of activity between neural regions or neurons. Functional networks of neuronal microcircuitry have been studied using various methods, such as microelectrode arrays, and have been shown to exhibit many nonrandom features such as small-worldedness (Downes et al. 2012; Watts and Strogatz 1998), well-defined community structure (Shimono and Beggs 2015), hubs (Shimono and Beggs 2015; Timme et al. 2016), and motifs (Song et al. 2005; Perin et al. 2011). The pre- and postsynaptic roles of connected neurons imply that information flow is directed in a neural network, so we only consider the directed case for both structural and functional networks. We note that directed functional connections, which signify causal influence, are frequently referred to as “effective connections.” In spite of our focus on directed connections in this paper, we use “functional” in place of “effective” throughout as our methods could also be applied to the undirected case.

While it is known that the functional network is influenced by the synaptic structure, it is not known whether the underlying synaptic connectivity has the same features of the functional network. A few computational studies have attempted to answer how closely the functional network may match the synaptic network from which it arises. In (Garofalo et al. 2009; Ito et al. 2011), the authors used random networks of Izhekevich neurons and various correlation measures to investigate the one-to-one matching of functional and structural networks. While (Garofalo et al. 2009) showed fairly poor matching between structural and functional networks, (Ito et al. 2011) used higher-order transfer entropy to functionally infer up to 80% of existing synaptic connections (true-positives) at low rates of false-positive occurrence (functional connections where no synaptic connection exists). In (Kobayashi and Kitano 2013), the authors furthered this direction by studying regular and small-world networks. The authors showed that as the probability for the creation of small-world connections increased, the one-to-one matching of the structural network and functional network decreased. These articles demonstrated that functional networks do not match exactly the underlying synaptic structure, but rather may include false-positives and false-negatives (an absence of functional connection where a synaptic connection exists). We investigate the nonrandomness of these false-positive (FP) and false-negative (FN) features to address the influence of structural on functional neural networks.

**Fig. 1:**
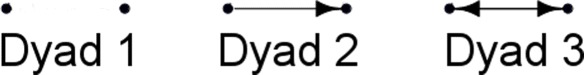
Dyadic Subgraphs. All nonisomorphic directed graphs consisting of two vertices. Dyad 3 represents recurrent connections which are thought to be important in information storage and processing in neural circuits

**Fig. 2:**
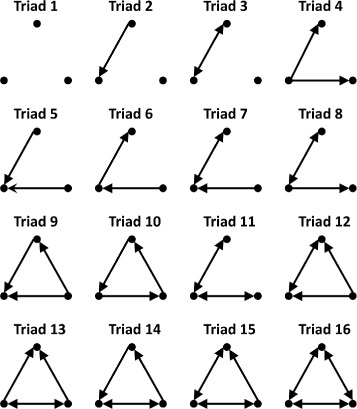
Triadic Subgraphs. All nonisomorphic directed graphs consisting of three vertices. Subgraphs that occur at statistically high rates are called motifs. It is common in literature to ignore subgraphs 1, 2 and 3; however, we include them as possible transformation results

A possible influence of the location of FP’s and FN’s may exist in the local synaptic connectivity, which can be represented as subgraphs of the larger network. Subgraphs that occur more often in a given network than in random models have been of particular interest. These overrepresented subgraphs are called motifs and may play an important role in network function (Milo et al. 2002; Alon 2007). In directed networks, motifs are directed subgraphs whose occurrence counts have high positive *Z*-scores when compared to a suitable random null model. Subgraph motifs are classified by the number of vertices they contain. Two types of subgraphs have been found to be particularly interesting for neural networks: dyadic (2-vertex) and triadic (3-vertex) (Shimono and Beggs 2015; Song et al. 2005; Perin et al. 2011; Guo and Li 2009; Li 2008; Vasilaki and Giugliano 2014). All nonisomorphic dyadic and triadic subgraphs are shown in Figs. 1 and 2 respectively. For microscopic neural networks, recurrent (reciprocal) synaptic connections, represented by dyad 3, have been hypothesized to allow for the storage of large amounts of information in neural circuits (Brunel 2016). These types of synaptic connections have also been found to be overrepresented in higher-order network motifs (Sporns 2010; Rubinov and Sporns 2010; Song et al. 2005), particularly in triadic motifs which have been studied in biological neural networks (Shimono and Beggs 2015; Perin et al. 2011; Song et al. 2005; Akin et al. 2016). Certain triadic motifs have also been found to have interesting functional roles and influence over local communication through clustering effects in neuronal networks (Guo and Li 2009; Li 2008). Given the aforementioned importance of dyadic and triadic structures in information-processing and storage, it is of interest to study how well these subgraph connections are captured in functional network reconstructions and their influence over FP and FN location.

We simulate structural networks of 100 neurons, 80% excitatory and 20% inhibitory for ten trials. These neurons are synaptically connected using a lattice network with small-world properties. Unlike (Ito et al. 2011; Kobayashi and Kitano 2013; Garofalo et al. 2009) we do not use a fully recurrent network but rather introduce unidirectional connections. We construct our network in such a way that we control the percentage of recurrent connections. We run these simulations for ten trials randomizing the small-world and recurrent connectivity and placement of inhibitory neurons. We then use a network of 1024 neurons with the same properties to ensure that our results scale up to larger networks. From these simulations, we obtain ten minute spike trains which we use to build our functional networks using two model free correlation measures.

We choose two correlation methods based on different ideas of how information is passed between neurons. At the microscopic scale, information is transferred from presynaptic to postsynaptic neurons via action potentials. It is unclear, however, whether information-processing in neural circuits is influenced more heavily by the rates of spiking (firing rates) or the temporal spiking patterns of the constituent neurons. As correlation methods account for these spiking properties differently, the chosen method will have a large role in determining functional topology. Here we use two well established methods, transfer entropy (Ito et al. 2011) and a Gaussian convolution method (Segev et al. 2004), to study the different spiking properties. Transfer entropy is based on spike pattern correlations while the Gaussian convolution method is based on firing rate correlations.

To study the change in topology from the structural to the functional network, we use a multilayer network (Boccaletti et al. 2014; Kivelä et al. 2014). Single layer networks, known as monoplex networks, are limited in that their edges represent single types of interaction. In contrast, multilayer networks have been increasingly used to accommodate multiple aspects of vertex interaction where each layer uses a different type of edge. Multilayer networks have found extensive application in areas of study such as social (Mucha et al. 2010; Klimek and Thurner 2013; Corominas-Murtra et al. 2014; Horvát and Zweig 2013; Battiston et al. 2014), economic (Lee and Monge 2011; Barigozzi et al. 2010; Barigozzi et al. 2011), and biological (Nicosia and Latora 2015; Li et al. 2012) interactions. Here, we apply multilayer network analysis to study the relationship between structural and functional networks and in particular to determine how the former influences the development of the latter. Specifically, we focus on the reconstruction of dyadic and triadic subgraphs in general by defining multilayer subgraph transformations from structural networks to functional ones.

Our results suggest that certain functional subgraphs arise from specific structural subgraphs with statistical significance. Specifically, we note high *Z*-scores corresponding to coupled structural and functional subgraphs that possess certain properties such as the presence of hubs. We discuss possible explanations regarding the presence of FP’s and FN’s within this context.

## Network model

Simulations of neurons on networks with different topologies have been used to study many aspects of neuronal activity including synchronization (Lago-Fernández et al. 2000; Han et al. 2013; Kim and Lim 2015), neuronal avalanches (Pajevic and Plenz 2009; Massobrio et al.^2015^) and functional reconstruction (Ito et al. 2011; Kobayashi and Kitano 2013; Hlinka and Coombes 2012; Orlandi et al. 2014). Here we use simulations of simple spiking models on small-world networks to study effective reconstruction of local connectivity. We set up networks of 100 neurons, 20 inhibitory and 80 excitatory, based on the Izhikevich spiking model (Izhikevich 2007; 2003). We start with a regular, fully recurrent (i.e. undirected) network, with connections based on distance between vertices. We then rewire connections randomly to create a small-world network in the manner of Watts and Strogatz Watts and Strogatz (1998). To reduce the number of recurrent synaptic connections, we randomly select a number of edges and delete one direction of those edges. This creates a network with small-world features and with a given percentage of non-recurrent edges. We then scale up to 1024 neurons to ensure that results hold for a larger number of neurons. The inclusion of small-world features is only for the sense of biological realism. We are not interested in studying the functional reconstruction of small-world features, but only of the dyadic and triadic subgraph motifs.

### Neuron models

We use the Izhikevich neuron model as it allows for a wide variety of spiking options while maintaining computational simplicity. The Izhikevich model is a resetting spiking model of the neuron voltage. When the potential reaches a threshold, it resets to a subthreshold level. We choose two basic neuronal parameter sets for our network: regular-spiking excitatory pyramidal cells and fast-spiking inhibitory interneurons (Izhikevich 2007). The pyramidal cell dynamics are given by

1 $$\begin{array}{@{}rcl@{}} 100\dot{v} &= &0.7(v+60)(v+40)-u+I_{E}\\ \dot{u} &=& 0.03(-2(v+60))-u  \end{array} $$

When the voltage is greater than or equal to 35mV, *v* is reset to -50mV and *u* is set to *u*+100. *I*_*E*_ is the summation of the synaptic inputs into the excitatory cells and an external random Poisson excitatory input. For inhibitory interneurons, we use the fast-spiking model given by

2 $$\begin{array}{@{}rcl@{}} 20\dot{v} &=& (v+55)(v+40) -u +I_{I}\\ \dot{u} &=& 0.2(U(v)-u)  \end{array} $$

For this particular model, when *v*≥25mV, *v* resets to -45mV. The adaptation variable *u* depends on a function *U*(*v*). This function *U*(*v*) is dependent on a threshold value of *v*_*b*_, which we set to -55mV. If *v*≥*v*_*b*_ then *U*(*v*)=0.025(*v*−*v*_*b*_)^3^, otherwise *U*(*v*)=0. *I*_*I*_ is the summation of the synaptic inputs into the inhibitory cells and a random Poisson excitatory input which has a frequency of 10Hz. We use a simple exponential decay model for both excitatory and inhibitory synapses given by

3 $$ S_{X} = v_{X}e^{\frac{t-t_{X}-t_{k}}{\tau}}  $$

where *X*∈{*E,I*}. *v*_*X*_ is the maximum voltage increase delivered by the synapse to the postsynaptic cell. For each excitatory synaptic connection, we assign a *v*_*E*_ randomly from a Gaussian distribution with mean 3.1 and standard deviation 0.1. All *v*_*I*_ are set to -1.5mV. For all synapses, the decay constant *τ* was set to 3ms. The time *t*_*X*_ was set to 5ms for excitatory synapses and 1ms for inhibitory synapses. The *t*_*k*_ variable represents the time that a spike occurs in the false-positive cells. Neural networks were simulated using Cython, a compilable extension of Python.

### Network construction

We arrange 100 neurons, 80 excitatory and 20 inhibitory, at random on a 10×10 grid initially with undirected connections between any pair of neurons separated by distance $\sqrt {2}$ or less. This gives a total of 342 undirected edges among interior neurons (eight incident edges), boundary neurons (five incident edges), and corner neurons (three incident edges). This regular lattice network structure does not have periodic boundaries and thus does not allow for propagation of looping activity through the network.

We use Watts and Strogatz Watts and Strogatz (1998) method to form a small-world network from the regular network. We define a probability, *p*_*rw*_ as the probability for an undirected edge to be rewired. This process creates long range connections within the network giving it small-world properties. For *p*_*rw*_=0 no edges are rewired and the network remains regular. For *p*_*rw*_=1 the network is completely random. This process creates long range connections within the network giving it small-world properties.

We then reduce the number of recurrent edges by randomly selecting a number of edges and deleting one direction of it’s recurrent connection. We set a probability *p*_*r*_ which determines the proportion of undirected edges in the network which are selected to become directed. Another probability *p*_*d*_ determines which direction is deleted from each chosen undirected edge. When *p*_*r*_=0, the network remains completely recurrent, and when *p*_*r*_=1 the network has no recurrent edges. We will always set *p*_*d*_=0.5 so that the direction of deletion has no preference. It is interesting to note that the network is acyclic when *p*_*r*_=1 and *p*_*d*_=0 or 1.

## Spike train analysis

We simulate a spiking network of Izhikevich neurons to obtain a spike time-series *s*_*i*_ for each neuron *i*. We then use two model-free correlation methods, transfer entropy (Ito et al. 2011) and a Gaussian convolution/Pearson Correlation method (Segev et al. 2004), to obtain dependencies between neuron pairs using the spike time-series. These methods are respectively representative of two theories that information-processing is influenced by (a) temporal spiking patterns and (b) spiking rates. These measures are then thresholded at multiple values to produce functional network reconstructions.

### Higher order transfer entropy

Transfer entropy (TE), a standard tool for functional network reconstruction, identifies directed functional connections by comparing the spike-trains in 1ms time bins among a group of recorded neurons. For a given pair (*i,j*) of neurons, TE is a measure between zero and one which is greater in magnitude when including the spiking history of neuron *j* better allows for an accurate prediction of the spiking behavior of neuron *i*. We adopt a version of higher-order transfer entropy (HOTE), introduced in (Ito et al. 2011), which accommodates TE computation over a range of delays between two spiketrains as well as over a range of orders; that is, the lengths of the spiking histories observed for neurons *i* and *j*. The HOTE formula is given by

4 $$ TE_{j \to i}(d)=\sum p\left(i_{t+1},i_{t}^{(k)},j_{t+1-d}^{(l)}\right)\log_{2} \frac{p\left(i_{t+1}|i_{t}^{(k)},j_{t+1-d}^{(l)}\right)}{p\left(i_{t+1}|i_{t}^{(k)}\right)}  $$

where *i*_*t*_,*j*_*t*_ give the states of neurons *i* and *j* (1 for the presence and 0 for the absence of a spike) at time bin *t*. *k* and *l* are the fixed orders of neurons *i* and *j*, respectively, and *d* represents the time delay between the observed states of the two neurons, ranging from 0 to 30ms. We set *k*=5 and *l*=5, and we compute HOTE using the Matlab toolbox developed by Ito’s group (Ito et al. 2011). We take use maximum value of TE over the delay *d*.

### Gaussian convolution correlation

In addition to transfer entropy, we used a Gaussian convolution-correlation method to compute information flow between neurons (Segev et al. 2004). This method is based on the idea that a neuron’s firing rate encodes information, therefore neurons with similar firing rate profiles encode common information. Convolution of our binned spike train *s*_*i*_ with a Gaussian kernel produces a continuous signal *x*_*i*_ whose amplitudes correspond to firing rates. For spike train *s*_*i*_, the convolution is given by

5 $$ x_{i}(t)= \int_{-\infty}^{\infty} s_{i}(\tau) G(t-\tau)d\tau  $$

where *G*(*t*) is a Gaussian with standard deviation *σ*=0.2 and mean zero. We compute the Pearson correlation coefficient given by,

6 $$ CC_{j\rightarrow i}(d) = \frac{\sum_{t=1}^{n}\left(x_{i}[\!t] -\bar{x}_{i}\right)(x_{j}[\!t-d] - \bar{x}_{j})}{\sqrt{\sum_{t=1}^{n} \left(x_{i}[\!t] -\bar{x}_{i}\right)^{2}}\sqrt{\sum_{t=1}^{n} \left(x_{j}[\!t-d] -\bar{x}_{j}\right)^{2}}},  $$

where $\bar {x}_{j}$ is the mean of *x*_*j*_ over *n* samples, *d* is the delay, and *x*_*i*_[ *t*] is the Gaussian convolution signal sampled at time *t* with 1ms steps. We add multiple delays into one spike train to identify directed information flow. For correlating neuron *j* to neuron *i*, our spike time delays range from 1 to 30ms with steps of 1ms for the *j* spike train. The maximum value of *CC*_*j*→*i*_(*d*) over *d* is taken as the correlation from neuron *j* to neuron *i*. Gaussian convolution and Pearson correlation were done in Python.

### Thresholding

To evaluate the significance of the reported TE (CC) result, each neuron in the network takes its turn serving as the “center” of the network, and we compute the mean *μ* and standard deviation *σ* of all TE (CC) values corresponding to connections which involve the chosen neuron. Since our correlations are all directed, this requires separate statistical calculations for the center neuron’s incoming and outgoing TE (CC) values. For a choice of parameter *κ* and center neuron *j*, we compute the outward (*j*→) and inward (*j*←) thresholds, $\gamma _{j \rightarrow }^{(\kappa)}$ and $\gamma _{j \leftarrow }^{(\kappa)}$, respectively, by

7 $$ \gamma_{j \rightarrow}^{(\kappa)}=\mu_{j\rightarrow}+\kappa \sigma_{j \rightarrow},\ \gamma_{j \leftarrow}^{(\kappa)}=\mu_{j \leftarrow}+\kappa \sigma_{j \leftarrow},  $$

and the directed correlation from neuron *j* to neuron *i* is *statistically significant with respect to* *κ* if and only if

8 $$ TE_{j \to i} \geq \max \left\{ \gamma_{j \rightarrow}^{(\kappa)}, \gamma_{i \leftarrow}^{(\kappa)} \right\}.  $$

Using this thresholding method, we create an asymmetric matrix *TE* ^(*κ*)^ of correlation values where $TE_{ij}^{(\kappa)} = TE_{j\rightarrow i}$ if *TE*_*j*→*i*_ is statistically significant with respect to *κ*, otherwise it is zero. CC is handled identically to create correlation matrix *CC* ^(*κ*)^. Thresholding code was written in Python.

## Multiplex network analysis

### Multiplex networks

The statistically significant correlations we find in the previous section are next used to build a functional reconstruction of the structural network. We employ multiplex networks to compare the structural and functional networks in order to analyze the influence of the former on the latter. Multiplex networks are special cases of multilayer networks for which all layers consist of an identical set *V* of vertices. Interlayer edges exclusively connect corresponding vertices, but the intralayer edges *E*_*i*_ of layer *i* may have different configurations across layers to represent different relationships among the set of verticess. For our study, each multiplex network *M* consists of two layers: the structural network *S*=(*V,E*_*s*_) and the functional layer *F*=(*V,E*_*f*_). We denote the multiplex network *M*={*S,F*}. The set of vertices consists of the 100 neurons which are treated identically rather than distinguished as excitatory or inhibitory. The set *E*_*s*_ are edges representing the connections from the structural network and *E*_*f*_ are edges representing statistically significant functional correlations. Since the functional correlations are dependent on the threshold *κ*, we can further specify *M* using *M* ^(*κ*)^.

In our study, it is useful to use adjacency matrices representing each layer of the multiplex network and also the supra-adjacency matrix of the entire multiplex network. An adjacency matrix is a matrix *A* with entries *a*_*ij*_∈{1,0} with *a*_*ij*_=1 if there is an edge from *j* to *i* and *a*_*ij*_=0 otherwise. Each layer of the multiplex network has its own adjacency matrix consisting of intralayer edges. We denote the structural and functional matrices by *A*_*s*_ and *A*_*f*_, respectively. The supra-adjacency matrix for the multiplex network is a block matrix given by


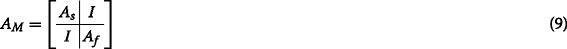


where *I* is the identity matrix. Since interlayer edges only connect each vertex to its copy across layers, we use the identity matrix for off-diagonal blocks.

### Functional subgraph transformations

We expect functional reconstructions to differ naturally from the underlying structural network, and each edge discrepancy is classified as either a false-positive (FP) or a false-negative (FN). FP’s are edges in the functional network that represent statistically significant correlations between neurons without synaptic connections. FN’s are synaptically connected neurons in the structural network whose correlation is below threshold and thus lack the corresponding edge in the functional network. These FP’s and FN’s can provide us with insight as to how information flows through the network and to how directed functional edges demonstrate the strength of directed information sharing.

**Fig. 3:**
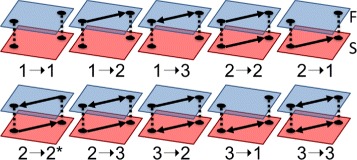
Dyadic Transformations. All ten nonisomorphic dyadic transformation multiplex subgraphs. We see preservations subgraphs being represented by 1→1, 2→2 and 3→3. 2→2^∗^ represents direction reversal of the 2 subgraph. All other transformations are conversion transformations

To study how the structural topology influences the functional topology, we define *functional transformations of structural subgraphs*, or briefly *functional transformations*, as multiplex subgraphs of the the *M* ^(*κ*)^ networks. These functional transformations allow us to analyze how structural connectivity informs corresponding functional connectivity. We focus on two types (dyadic and triadic) of structural subgraphs and their functional transformations. Dyadic subgraphs are subgraphs of a network consisting of exactly two vertices (neurons) and are shown in Fig. 1. A *dyadic functional transformation* is then defined by the multiplex subgraph consisting of two structural vertices, their functional layer counterparts, and the intralayer and interlayer edges. These thus form multilayer subnetwork with a 4×4 supra-adjacency matrix with the diagonal blocks being the structural and functional subgraph adjacency matrices and the off-diagonal blocks being identity matrices. Examples of these transformations are seen in Fig. 3. While there are exhaustively sixteen such transformations, we focus only on the ten given in the figure as the remaining six are each isomorphic to one of these ten. We define *preservation (conversion) transformations* as those which contain identical (distinct) subgraphs in each layer. Note we denote a transformation from structural subgraph *a* to functional subgraph *b*, where *a,b* are subgraph labels, as *a*→*b*.

Triadic subgraphs are subgraphs of a network consisting of three vertices and their edges. All sixteen are shown in Fig. 2. We use triadic subgraphs in the structural network along with their corresponding functional reconstructions to study how the surrounding local circuitry may influence FP and FN location. From this, we can determine which subgraphs may promote local information flow and which may be prone to information loss.

**Fig. 4:**
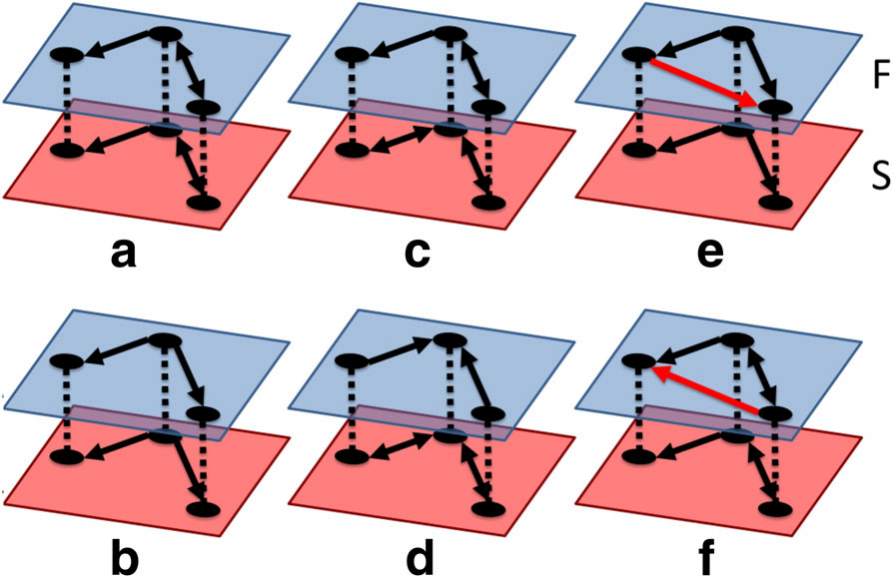
Triadic Transformations. Examples of triadic transformations. (**a**) and (**b**) show triad-preserving transformations for triads 8 and 4, respectively. (**c**) shows the transformation from triad 11 to triad 8 and (**d**) is the transformation from triad 11 to triad 5. (**e**) transforms triad 4 to 9 and (**f**) transforms triad 8 to 13. Both (**e**) and (**f**) demonstrate the addition of a false-positive (FP) (red edges are FP). All vertices are treated as identical

We define *triadic functional transformations* in the same manner as the dyadic case, with three neurons per layer. These then form 6×6 supra-adjacency matrices in the same manner as the dyadic transformations. Examples of triadic functional transformations are shown in Fig. 4. We study the 256 pair transformations of the 16 triadic subgraphs. These transformations represent the change in local topology from the structural to the functional level. As our functional networks represent information flow throughout the network, these transformations also indicate how well information is preserved in local circuits of neural networks. By comparing observed triadic counts with those of appropriate null models, we can determine whether observed FP’s and FN’s are nonrandom within triadic subgraphs. Observing the location of FP’s and FN’s within over- and underrepresented triadic subgraphs will aid us in determining local effects on information flow.

### Null models

In order, firstly, to demonstrate that the functional reconstruction is generated nonrandomly from the underlying structural network and, secondly, to determine how structural topology influences deviations in the functional network, we devise two null models. From these, we compute *Z*-scores, which measure overrepresentation (high *Z*-score) and underrepresentation (low *Z*-score) of the functional transformations observed in our networks. The first null model will compare our multiplex network to a null version whose structural layer has been randomized in order to convince us that the underlying structural layer nonrandomly influences the functional reconstruction. *Z*-scores of dyadic functional transformations will then be computed to determine if the reconstruction is statistically similar to the structural network. We focus on dyadic transformations as these pertain to the interactions between each distinct pair of neurons. Statistically overrepresented preservation transformations and underrepresented conversion transformations indicate a closer match between the structural network and the functional reconstruction. Such a match suggests that the structural network has high influence over the functional reconstruction. If the transformation *Z*-scores are close to zeros, the reconstruction is near random, indicating that the structural connectivity does not influence the functional reconstruction.

The second null model will inform on the contributions of local circuitry to deviations of the functional network from the structural network. To create it, we will randomize the FP’s and FN’s in the functional network. Using this null model and the *Z*-scores of triadic functional transformations, we can determine if the local structural topology affects the functional location of FP’s and FN’s. Again, we look for transformations with high and low *Z*-scores as these deviations from random reconstruction can inform us as to how the local structure influences functional reconstruction and information flow.

For notational convenience, we will omit the threshold parameter *κ* in the following sections. It should be understood that each functional network *F* and hence each multiplex network *M* depends upon the choice of *κ*.

#### Null model one

We build our first null model by randomizing the structural network *S* while maintaining the in, out, and recurrent degree sequences. For statistical significance, we randomize *S* for *N* instances creating the set of networks $\{ S_{i}^{r} \}_{i=1}^{N}$. The mean and standard deviation of functional transformation counts will be used to compute the *Z* score.

To randomize the structural network, we use the following algorithm. First, we randomly permute the vertices of the network to create *S*_*p*_ (with adjacency matrix *A*_*p*_) by applying a random permutation matrix *P* to the adjacency matrix of the structural network *A*_*s*_ via

10 $$ A_{p} = P^{-1}A_{s} P  $$

However, randomization in this manner is insufficient as *S*≃*S*_*p*_ and thus has many of the same properties. To further randomize the network, we rewire edges of the network in such a way as to maintain the in, out and recurrent degree sequences. To do this, we randomly select two edges, either both recurrent {{*a,b*},{*c,d*}} or both unidirectional {(*a,b*),(*c,d*)}, where *a,b,c,d* are vertices. We only treat directed edges as ordered pairs since recurrent connections are handled as undirected edges. We then rewire the edges by swapping vertices *a* and *c*, maintaining which vertex is a source and which is a sink in the directed case. If the randomly selected edges contain any vertex in common, the edges are discarded and the process is repeated for 100 iterations. This new network is our randomized structural network $S_{i}^{r}$. Each $S_{i}^{r}$ is then paired with the fixed functional network to obtain a set of *N* multiplex networks $\{ M_{i}^{rs} \}_{i=1}^{N}$ where $M_{i}^{rs}=\{ S_{i}^{r},F \}$.

We then count the number of dyadic transformations observed in each of the *N* multilayer networks and find the average *μ* ^*rs*^ and standard deviation *σ* ^*rs*^ of the counts of each transformation. We compute the *Z*-score of each transformation as captured by the given correlation method using

11 $$ Z(l)=\frac{l-\mu^{rs}}{\sigma^{rs}}  $$

where *l* is the transformation count from *M* ^(*κ*)^.

#### Null model two

As our focus is on how local connectivity affects the location of FP’s and FN’s in multilayer triadic subgraphs, we need a null model that will randomize the location of these FP’s and FN’s within the multiplex network. This randomization would be what is expected if local connectivity played no role in the locations of FP’s and FN’s. To create this model, we copy the structural network *S* and randomly delete (add) edges corresponding to the numbers of FN’s (FP’s) inferred in *F*. We add FP’s in places where there are no edges in *S* and FN’s only where edges exist in *S*. only We denote the resulting network $F^{r}_{i}$, and *N* such randomizations produce the set $\{ F_{i}^{r} \}_{i=1}^{N}$. We generate a set $\{ M_{i}^{rf} \}_{i=1}^{N}$ of new multiplex networks $M^{rf}_{i} = \{S,F^{r}_{i}\}$ by pairing each $F_{i}^{r}$ with the original structural network *S*. As before, we count the transformations across $\{ M_{i}^{rf} \}$ to obtain a mean *μ* ^*rf*^ and a standard deviation *σ* ^*rf*^ for the counts of each transformation. We calculate the *Z*-score of each triadic transformation by replacing (*μ* ^*rs*^,*σ* ^*rs*^) in the previous formula with (*μ* ^*rf*^,*σ* ^*rf*^). The result will indicate whether or not FP and FN functional edges are more likely to arise from certain triadic subgraphs of the structural network, suggesting influence from local connectivity schemes on the information flow within the network. Both null models were created using Python.

## Results

We simulate 10 networks of 100 neurons with *p*_*r*_=0.4, which makes 60% of synaptic connections recurrent, and *p*_*rw*_=0.4. Each trial is a different randomization of long distance (small-world) connections and recurrent connections. From these 10 trials, we obtain an average and standard deviation of the counts and *Z*-scores of our dyadic and triadic transformations. We use this information to demonstrate that the functional reconstructions are nonrandom and to determine how the local topology of the structural network informs that of the functional network. For our null model studies, we use *N*=100 randomizations for each trial.

### Dyadic counts

**Fig. 5:**
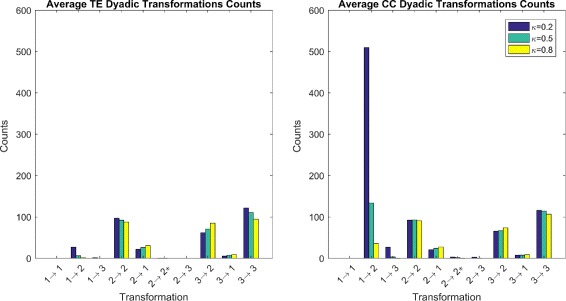
Average Dyadic Transformation Counts. Average dyadic transformation counts for TE and CC methods over all 10 trails. We notice the significantly higher number of counts for FP involving transformations 1→2 and 1→3 in CC compared to TE. We also note the rapid loss of these two transformations as *κ* increases. Most other transformation counts are similar at all thresholds for CC and TE

We first look at counts of the dyadic transformations of both the transfer entropy and Gaussian reconstruction methods as a comparison of the two measures TE and CC. We ignore the trivial preservation transformation 1→1 as this count is of no real interest for this analysis. The average of counts for both CC and TE are shown in Fig. 5. We immediately notice that at all thresholds, the CC method has a significantly higher number of FP transformation 1→2. This is most likely due to the linearity of the Pearson correlation, which results in higher correlations between neurons. To adjust for this, we may want to use higher thresholds for the CC method; however, this may be ill-advised as, on the other hand, the counts of preservation transformations for CC and TE are similar to one another at corresponding thresholds. Increasing the threshold parmeter *κ*, leads to a higher number of FN’s than we would like. As such, choosing the threshold will have significant influence on the topology of the functional network. Here we stick to thresholds with *κ*∈{0.2,0.5,0.8} as they give a high number of true-positives; that is, functional correlations above threshold when a synaptic connection exists.

### Functional reconstruction

**Fig. 6:**
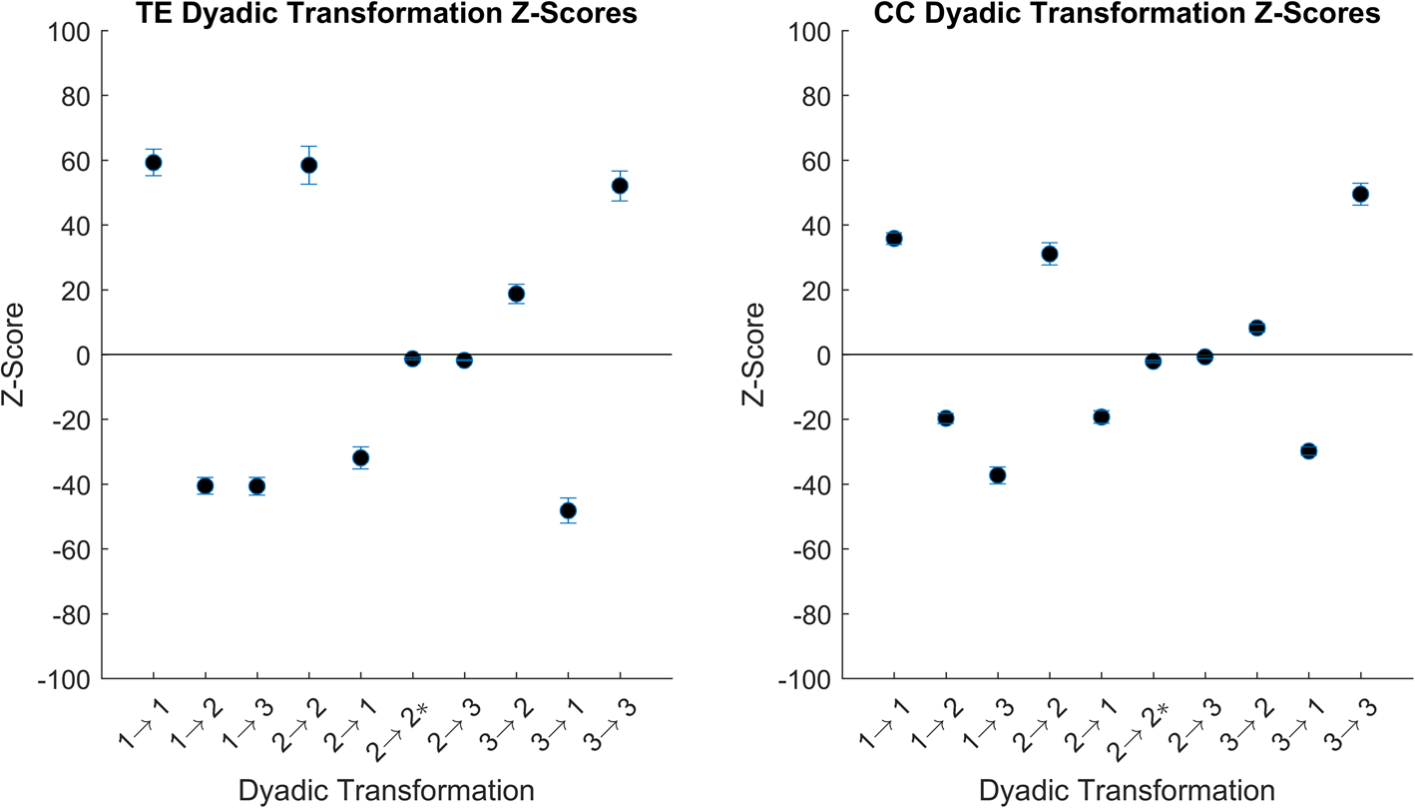
Dyadic *Z*-scores for *κ*=0.2. Average and standard deviations of *Z*-scores for both TE and CC methods with *κ*=0.2. We note that transformations 2→2^∗^ and 2→3 are close to zero while all other transformations are far from zero. All preservation transformations, 1→1, 2→2 and 3→3 are overrepresented while conversion transformations, aside from the two previously mentioned, are underrepresented. All transformations exhibit low standard deviations

**Fig. 7:**
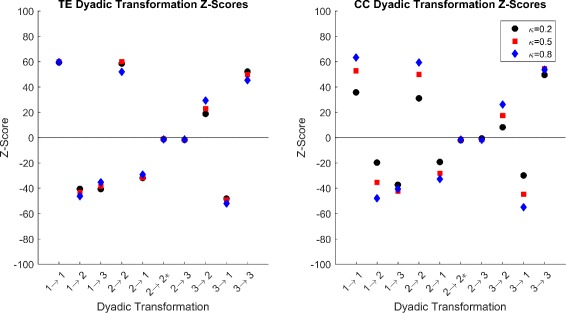
Dyad *Z*-score over all *κ*. Average dyadic *Z*-scores for *κ*∈{0.2,0.5,0.8} for CC and TE. We see that CC *Z* scores have more variance over thresholds than do TE. We note that CC thresholds are closer to zero at low *κ* than TE. As *κ* increases, CC *Z*-scores come closer to TE *Z*-score values. For all thresholds, 2→2^∗^ and 2→3 are close to zero and the markers overlap

We can infer the statistical significance of the extent to which the structural network influences the functional reconstruction using the first null model and the *Z*-scores of the dyadic transformations. The average *Z*-scores with standard deviations of the 10 dyadic transformations with *κ*=0.2 are shown in Fig. 6 for both TE and CC. Deviations for other *κ*, not shown, are similar or smaller. The majority of *Z*-scores show high deviations from random (*Z*>>0 or *Z*<<0), and we can conclude that the underlying structural network plays an important role in the topology of the functional network. This, of course, is intuitive; however, the choice of correlation method implemented affects the resulting *Z*-scores. The *Z*-scores of the CC method are closer to zeros as low threshold, Fig. 7 than TE. As threshold increases, CC *Z*-scores approach the near the sme value as TE *Z*-scores. This is most likely due to the increased number of FP’s seen in the CC reconstructions hence the larger edge density; that is, the number of edges divided by the total number of possible edges. A higher edge density will result in increased pairings through randomization, raising the mean and thus bringing the *Z*-score closer to zero.

We notice that two transformations 2→{2^∗^,3} remain near random regardless of threshold. Both these transformations have very low counts in in all cases of the network reconstruction due to the fact that they involve a functional edge directed in the opposite direction of the synaptic connection. It is expected that this would be rare as the correlation in the direction of the synaptic connection would dominate any correlation in the opposite direction. Due to the low *Z*-score of these transformations, we can treat functional edges occurring in such cases as random and not influenced by the underlying structure.

In the case of the preservation transformations, all *Z*-scores are positive and far from zero indicating strong influence from the structural network. As the thresholds increase, FP’s vanish at a faster rate than do TP’s, and the *Z*-scores increase indicating that the FP’s are important in determining how close to random our transformation *Z*-scores are. For conversion transformations representing FP’s and FN’s, we see negative *Z*-scores with large distance from zero. This indicates that FP’s and FN’s are more underrepresented than expected if reconstruction was random. From these results we conclude, as expected, that the reconstruction was nonrandom but rather highly dependent on the underlying structural network.

### Triadic subgraph influence on FP’s and FN’s

**Fig. 8:**
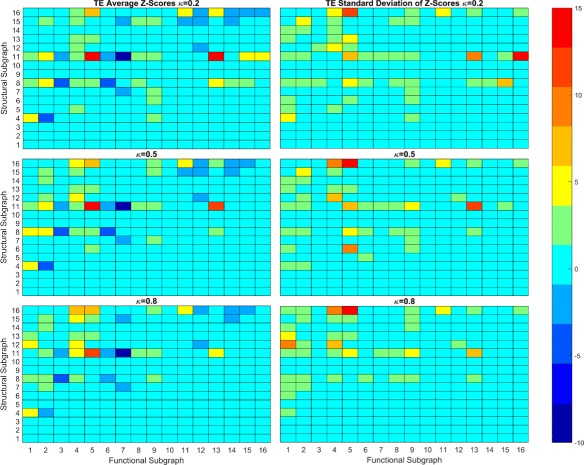
Average and Standard Deviation of Z-Scores for TE. Heatmap of average and standard deviation Z-scores of triadic subgraphs for TE reconstruction. The anti-diagonal consists of preservation transformations while off anti-diagonal are conversion transformations. The lower right triangle consists primarily of FP transformations while the upper left triangle consists primarily of FN transformations

**Fig. 9:**
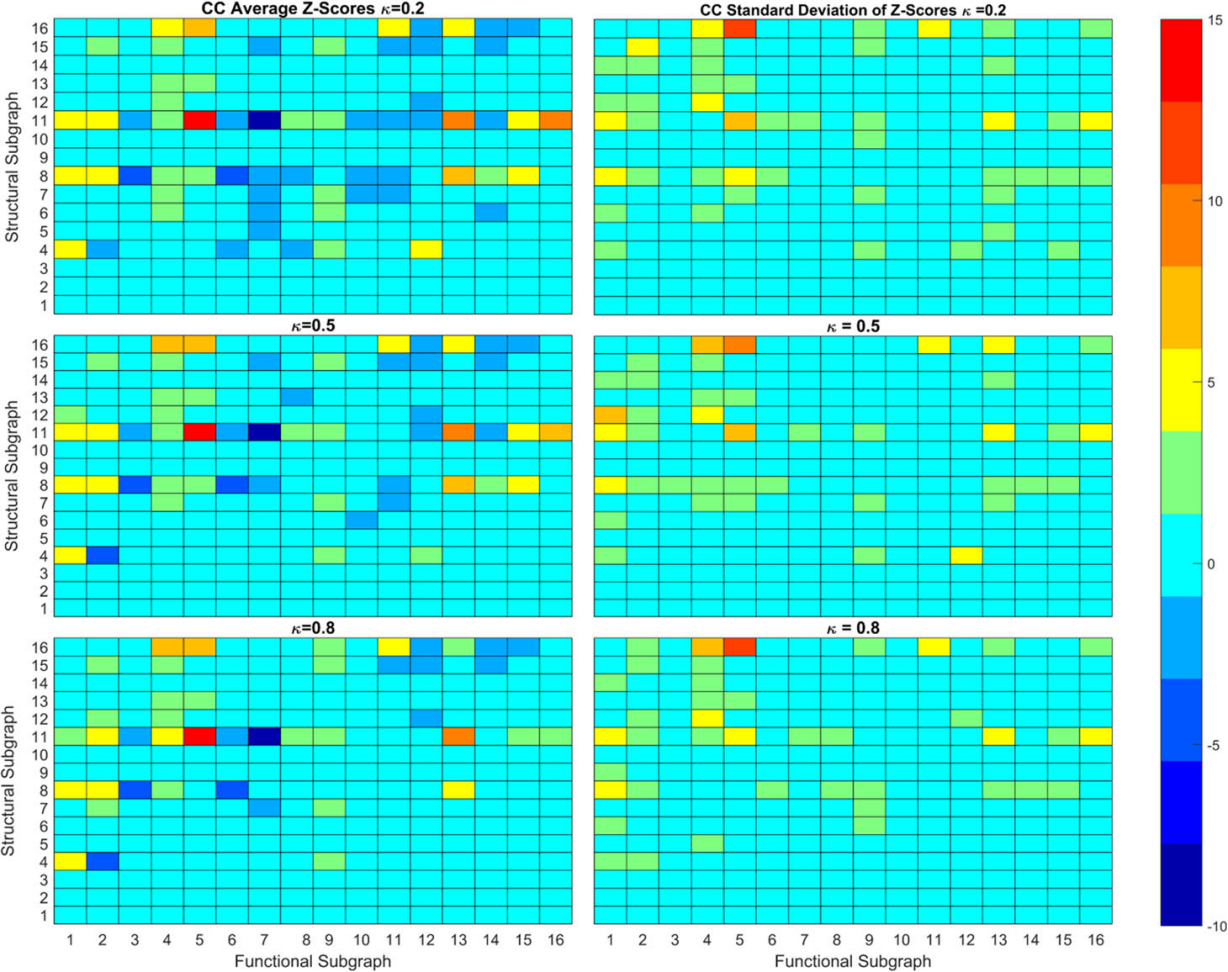
Average and Standard Deviation of Z-Scores for CC. Heatmap of average and standard deviation Z-scores of triadic subgraphs for TE reconstruction

We use the second null model to study how the local synaptic connectivity, in the form of triadic subgraphs, influences FP’s and FN’s in the functional reconstruction. Due to the high number of distinct triadic subgraph transformations, we use a heatmap of *Z*-scores Figs. 8 and 9. This gives a discrete heatmap with 256 blocks representing the transformations with preservation transformations on the diagonal. Here, we study transformations involving primarily FP’s and FN’s separately. Structural subgraphs {1,2,3} were ignored.

#### False positive

Transformations involving FP’s that occur between two neurons without direct synaptic connection occupy the lower right triangle of our heatmap. Since these types of FP’s are the only significant ones from our dyadic study, we focus on these here. As can be seen in Fig. 8, the TE heatmap contains fewer significant transformations in the lower triangle than the CC heatmap Fig. 9. This indicates that FP transformations have more influence over the CC reconstruction topology at the given threshold levels than in the TE reconstruction. As *κ* increases, we see that the number of significant FP transformations decreases as the number of FP’s decreases. While there is a difference in the number of transformations that are significant in both methods, we note similarities in which structural subgraphs produce significant transformations. For both reconstruction methods, the most significant transformations involving FP’s come from structural subgraphs {4,6,7,8,11}. This set consists of all subgraphs, outside of 5, that involve interactions of two of the three pairs of neurons within each triad. Not surprisingly, the addition of the FP occurs between the non-interacting pair of neurons. We note a few interesting aspects of these transformations.

All {4,6,7,8,11} subgraphs contain one of two subgraphs, a “chain” represented by 6 or a “hub” structure represented by 4. In subgraph 6, one neuron has a synapse directed to a second neuron which has another synapse directed to the third neuron forming a chain. In subgraph 4, one neuron has out-degree 2 and the other two have in-degree 1 and out-degree 0, therefore the neuron with out-degree 2 plays the role of a hub neuron in the subgraph. Subgraph 6 is a subgraph of 7, 8 and 11 while 4 is a subgraph of 8 and 11 only.

We notice that the transformations {4,6,7,11}→9 are overrepresented in both TE and CC reconstructions and have small standard deviations. This transformation from subgraph 6 indicates a FP in the direction of the chain; the FP originates at the first neuron in the chain and terminates at the last neuron. In this sense, the FP follows the information flow of the local circuit, and the first neuron has distant influence over information-processing. For the transformation 4→9, it is unclear if there is a preference in direction of the FP as reversing the FP would create an isomorphic transformation. We can speculate that the direction of the FP would be toward the neuron with which the hub neuron has a weaker synapse. The weaker synapse would influence spiking behavior but with a lag compared to the neuron with which the hub has a stronger synapse. Further study may be needed taking into account edge weights.

For transformations {7,11}→9, it is unclear what determines the direction of the FP due to a concurrent FN. For 7, it is reasonable to speculate that the FP will follow the chain structure as 7 does not have 4 as a subgraph. 7 does have 5 as a subgraph, but 5 is not implicated in FP transformations. Structural subgraph 11 has both 4 and 6 as subgraphs and thus we cannot conclude if the FP has a preferential direction.

Only subgraphs {4,8,11}, each containing the hub structure, are implicated in FP transformations to subgraphs other than 9. The transformation 4→12 occurs in CC reconstructions at low thresholds and indicates the addition of two FP’s between the neurons without synaptic connections. We note that this transformation has a somewhat high standard deviation, indicating higher variance trial to trial, but it is still at least somewhat overrepresented in all trials. Subgraph 8 transforms to subgraphs {13,14,15}, with 8→13 being more overrepresented than both 8→{14,15}. This indicates it is more likely that a FP will follow the chain subgraph than not. The transformation 8→13 involves the addition of a FP that follows the direction of the chain subgraph, while for 8→{14,15}, the FP direction is not clear. Note that while 8→{13,15} generally have high standard deviations, they were overrepresented in all trials at low thresholds. 8→15 indicates two FP’s are created in between the two non-interacting neurons which may be due to interactions between the chain subgraph and hub subgraph. Subgraph 11 transforms to subgraphs {13,15,16} at significantly high rates. As 11 contains two chain subgraphs and a hub subgraph, it is unclear what influences the FP direction and information flow. Synaptic strength studies may be enlightening here.

#### False negatives

FN transformations are contained mainly in the upper left triangle of the heatmaps. As we noted before, FP’s and FN’s may coexist within a single transformation. For our analysis here, we restrict ourselves to the structural subgraphs {8,11,16} as these are involved in a large number of significant FN transformations.

We see that all three have overrepresented transformations to 4 and 5 while transformations to 6 are underrepresented or random. However, from this it is not clear where the FN’s occur and whether or not there are FP’s involved. The transformation 8→5 must contain a FP as 5 is not a subgraph of 8. As such we can not determine what structural subgraphs may be most important for these transformations from this analysis. We note that transformation 11→7 is underrepresented while the transformation 11→8 is overrepresented. This seems to indicate that the 8 subgraph of 11, which contains the hub structure of 11, is a more robust subgraph than the 7 subgraph. Similarly, subgraph 16 transforms to subgraphs 11 and 13 at significant rates. The transformation 16→11 indicates a functional loss of a recurrent connection while 16→13 is a functional loss of a hub structure. It is interesting that 16→12 is not significant while 16→13 is, as the difference is the functional loss of a 5 or 4 subgraph respectively. This may be indicated by the transformation 4→1 being overrepresented and 4→2 being underrepresented. This indicates that it is more likely for 4 to have functional loss of both edges than just one. Note that some transformations have high standard deviations and that not all were seen in every trial. This may be overcome with larger networks as mentioned in the discussion.

### Larger network

**Fig. 10:**
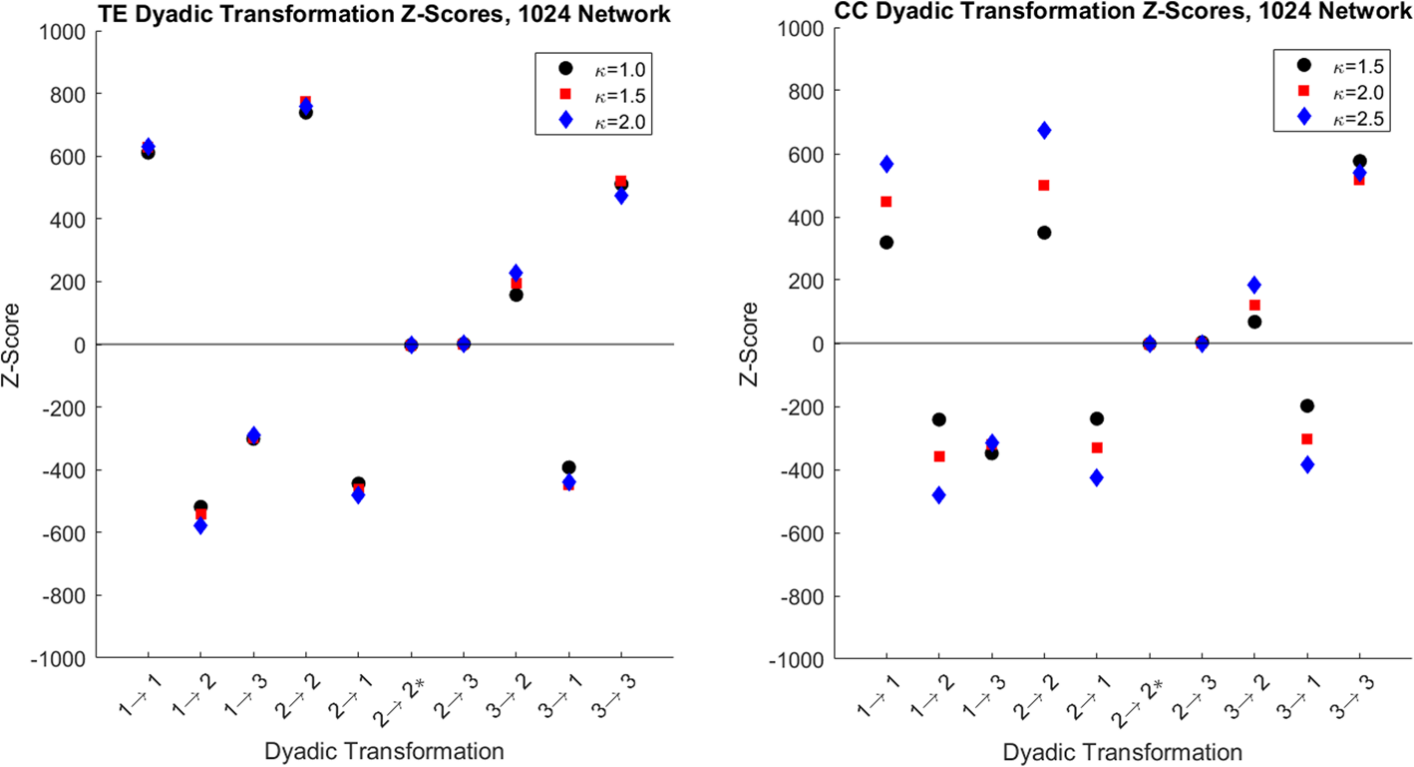
Dyadic *Z*-scores for 1024 neuron network. Shown are the *Z*-scores for both the TE and GC reconstructions. While the pattern from the smaller network is the same, we note that for both cases, the magnitude of *Z*-scores are significantly higher. We also use different values of *κ* for this network than the 100 neurons network

**Fig. 11:**
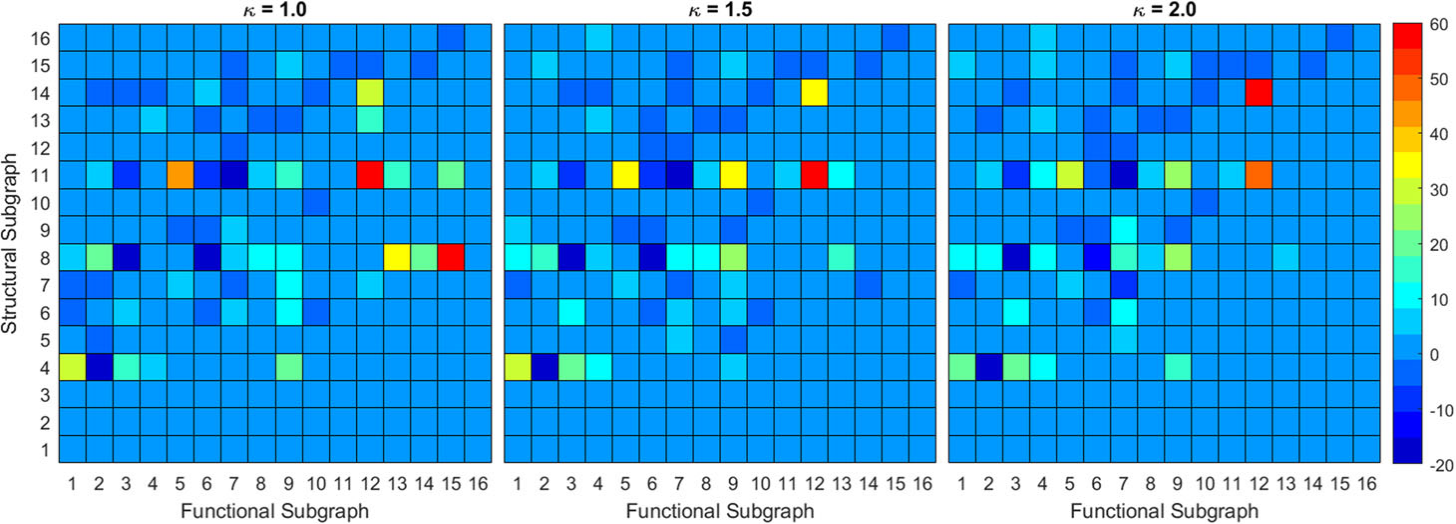
Triadic *Z*-scores for TE reconstruction of the 1024 neuron network. Heatmap of the *Z*-scores of the larger network using transfer entropy for functional reconstruction. The anti-diagonal consists of preservation transformations while off anti-diagonal are conversion transformations. The lower right triangle consists primarily of FP transformations while the upper left triangle consists primarily of FN transformations

**Fig. 12:**
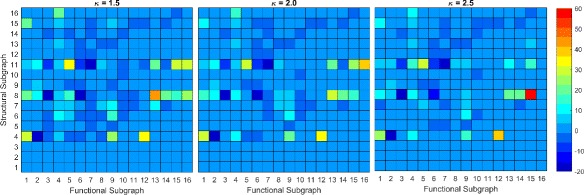
Triadic *Z*-scores for CC reconstruction of the 1024 neuron network. Heatmap of the *Z*-scores of the larger network using the Gaussian correlation method for functional reconstruction

We use a network of 1024 neurons with the same connectivity scheme to check whether our results scale up with network size. The *Z*-scores are shown in figure Fig. 10 for dyadic and Figs. 11 and 12 for triadic transformations. These results suggest patterns similar to the case of the smaller network. The dyadic *Z*-scores for the larger network imply overrepresentation of preservation transformations and underrepresentation of FP and FN transformations. The transformations 2→2∗ and 2→3 are still near zero and have near the same magnitude as for the 100 neuron network.

The results for triadic transformations similarly coincide with those obtained from the smaller network. The same structural triads are involved in FP transformations for both networks. The most significant transformations with the highest *Z*-scores involve the hub type structure. While the chain structure is still involved in transformations that have high *Z*-scores, the magnitude of the *Z*-score is lower, indicating that the chain structure, while important, is not as significant as the hub structure in determining effective network topology. We note the similar triadic *Z*-scores on the upper triangle for FN transformations in the 1024 neuron network as in the 100 neuron network. Thus, our main results from the 100 neuron network scale up to the larger network. This shows high influence of the structural topology on the effective network reconstruction.

There are some noteworthy differences between the 100 and 1024 neuron networks. A change in network size necessitates revision of our choices of *κ*. When we increase the number of neurons, the mean and standard deviation of the correlations decrease due to the increased number of neuron pairs without synaptic connections and, consequently, lower correlation values. Because of this effect, we need larger *κ* values in order to keep the number of FP’s low for a meaningful analysis of the larger network. The number of TP’s at larger *κ* values remains high and in proportion with the case of the smaller network.

Additionally, as the number of neurons increases, we would expect that the significant transformations remain the same while the magnitude of their *Z*-scores will increase. Intuitively, a larger network contains a greater number of triads which allow for more instances of each transformation, but these transformation counts remain low in the null models. This phenomenon is observed for both the dyadic and triadic cases.

In its current form, our graph search algorithm is not optimized and is prohibitively slow for much larger networks. As such only a single trial for the larger network was studied. Further optimization of the algorithm will allow for more extensive work with large networks.

## Discussion

In this paper, we have presented a study of how local network topology of spiking neural networks influences functional reconstruction using two model free methods. We used two methods that were based on different ideas of correlation between neurons; spike patterns with TE and neuron firing rate with CC. We used the directed correlations produced by each to determine information flow. As CC is a more coarse measure of correlation, we saw a higher number of FP’s in CC functional networks as opposed to TE functional networks. This result implies that some neurons have more influence over neurons with which they do not directly synapse. FN’s occurred in similar numbers in both TE and CC.

As FP’s and FN’s can inform us as to how information is processed in a network, we used multiplex networks to identify functional edges of this nature. We defined dyadic and triadic transformations to be multilayer subgraphs of our multiplex networks and used null models to determine the statistical significance of transformations. Using dyadic transformations, we showed that functional reconstruction for both TE and CC were highly dependent on the structural network and that FP’s are only significant when located between neurons without direct synaptic connection. We also showed that certain structural triadic subgraphs, the chain and hub structures, were likely to produce FP’s and that the direction of the information flow for the FP is determined by the structure. As these structures allow for information flow between neurons that are not connected, they may be biologically beneficial, allowing significant communication between neurons without the cost of synapses.

This may be illustrated by subgraph 11, which has been shown to be overrepresented in many biological neural networks (Sporns 2010). This subgraph contains a hub structure as well as two chain structures. We saw in this work that this subgraph was involved in more significant transformations than any other subgraph and thus was more influential over functional reconstruction.

We also showed that FN’s are influenced by the structure, but the cause of the influence is not clear from this study. More detailed analysis of the nonisomorphic transformations and the synaptic strengths of the structural network may be needed to determine why certain transformations are significant. At present, this problem has no easy solution due to the high number of transformations and the variance of synaptic strengths over structural subgraphs.

We used a large network with 1024 neurons and performed analysis over the whole network to test whether our results hold for larger networks; however, in most studies involving neural recordings, such as MEA studies, recordings are not obtained for each individual neuron in the network. We are therefore interesting in studying how sampling a given number of neurons from the larger network would affect the functional reconstruction. We hypothesize that the results would change significantly with a random sample from a larger network and would change based on the size of the sample. We think that results would change since a sample would not capture all the local connectivity. For instance, two neurons from the hub triad may be selected causing a false-positive that is not seen as part of a hub. We thus expect differences between significant transformations of a sample set and the whole network.

It has been shown in (Li 2008) that the position of inhibitory neurons within a triadic subgraph affects spiking behavior and consequently dependence within the triad. In this paper, we did not consider the position of inhibitory neurons in dyadic or triadic subgraphs. As such, further investigation into its effects on the functional reconstruction of these subgraphs may be useful. However, as vertices will not be identical in this case, the number of transformations increases dramatically making analysis much more difficult.

In our study, we used a fairly narrow Gaussian distribution of synaptic strengths which may not be realistic. To further our study, we can use either a wider Gaussian distribution or use spike-timing dependent plasticity (STDP) to learn synaptic strengths in order to obtain a less narrow distribution. This in turn will influence reconstruction of the structural network through an increase or decrease in dependence. It would also be of further interest to use STDP to study whether there is a learned preference for recurrent connection and certain triadic subgraphs.
